# [μ-Bis(diphenyl­arsino)methane-1:2κ^2^ *As*:*As*]deca­carbonyl-1κ^3^ *C*,2κ^3^ *C*,3κ^4^ *C*-*triangulo*-triosmium(0)

**DOI:** 10.1107/S1600536812024208

**Published:** 2012-07-25

**Authors:** Omar bin Shawkataly, Imthyaz Ahmed Khan, Siti Syaida Sirat, Ching Kheng Quah, Hoong-Kun Fun

**Affiliations:** a Chemical Sciences Programme, School of Distance Education, Universiti Sains Malaysia, 11800 USM, Penang, Malaysia; b X-ray Crystallography Unit, School of Physics, Universiti Sains Malaysia, 11800 USM, Penang, Malaysia

## Abstract

The title compound, [Os_3_(C_25_H_22_As_2_)(CO)_10_], contains a triangle of singly bonded Os atoms; both arsine ligands are equatorial with respect to the Os_3_ triangle. Each arsine-substituted Os atom bears one equatorial and two axial terminal carbonyl ligands, whereas the unsubstituted Os atom bears two equatorial and two axial terminal carbonyl ligands. The dihedral angles between the two benzene rings in the diphenyl­arsino groups are 67.42 (16) and 61.99 (16)°. In the crystal, mol­ecules are linked *via* C—H⋯O hydrogen bonds into zigzag chains propagating along [010].

## Related literature

For general background to *triangulo*-triosmium compounds with general structure of *M*_3_(CO)_12-_ *_n_L_n_* (*M* = Ru, Os and *L* = group 15 ligand), see: Bruce *et al.* (1985 ▶, 1988*a* ▶,*b* ▶); Shawkataly *et al.* (1998 ▶, 2004 ▶, 2010 ▶). For the preparation of the title compound, see: Filby *et al.* (2006 ▶). For the stability of the temperature controller used in the data collection, see: Cosier & Glazer (1986 ▶).
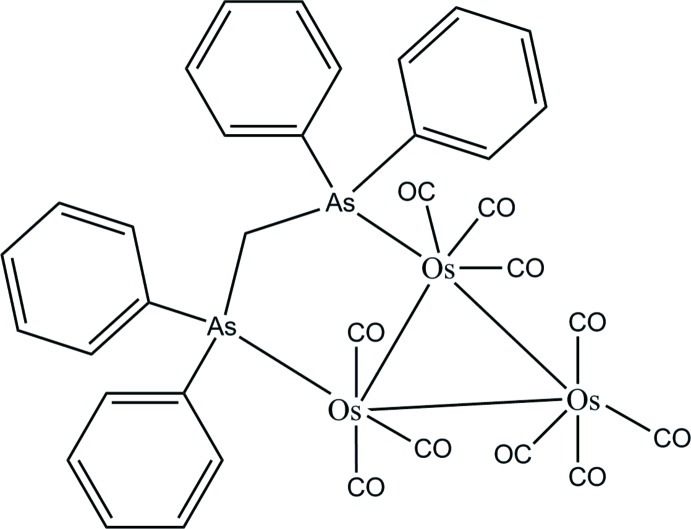


## Experimental

### 

#### Crystal data

[Os_3_(C_25_H_22_As_2_)(CO)_10_]
*M* *_r_* = 1322.97
Orthorhombic, 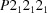

*a* = 11.2965 (4) Å
*b* = 15.3594 (5) Å
*c* = 20.6641 (7) Å
*V* = 3585.4 (2) Å^3^
*Z* = 4
Mo *K*α radiation
μ = 12.49 mm^−1^
*T* = 100 K
0.28 × 0.19 × 0.08 mm

#### Data collection

Bruker SMART APEXII DUO CCD diffractometer
Absorption correction: multi-scan (*SADABS*; Bruker, 2009 ▶) *T*_min_ = 0.126, *T*_max_ = 0.424
113235 measured reflections
15930 independent reflections
15315 reflections with *I* > 2σ(*I*)
*R*_int_ = 0.038

#### Refinement

*R*[*F* ^2^ > 2σ(*F* ^2^)] = 0.018
*wR*(*F* ^2^) = 0.040
*S* = 1.03
15930 reflections
451 parameters
H-atom parameters constrained
Δρ_max_ = 2.15 e Å^−3^
Δρ_min_ = −0.66 e Å^−3^
Absolute structure: Flack (1983 ▶), 7211 Friedel pairs
Flack parameter: 0.003 (4)

### 

Data collection: *APEX2* (Bruker, 2009 ▶); cell refinement: *SAINT* (Bruker, 2009 ▶); data reduction: *SAINT*; program(s) used to solve structure: *SHELXTL* (Sheldrick, 2008 ▶); program(s) used to refine structure: *SHELXTL*; molecular graphics: *SHELXTL*; software used to prepare material for publication: *SHELXTL* and *PLATON* (Spek, 2009 ▶).

## Supplementary Material

Crystal structure: contains datablock(s) global, I. DOI: 10.1107/S1600536812024208/hb6822sup1.cif

Structure factors: contains datablock(s) I. DOI: 10.1107/S1600536812024208/hb6822Isup2.hkl

Additional supplementary materials:  crystallographic information; 3D view; checkCIF report

## Figures and Tables

**Table 1:** Selected bond lengths (Å)

Os2—As1	2.4400 (3)
Os3—As2	2.4274 (3)

**Table 2:** Hydrogen-bond geometry (Å, °)

*D*—H⋯*A*	*D*—H	H⋯*A*	*D*⋯*A*	*D*—H⋯*A*
C16—H16*A*⋯O4^i^	0.93	2.53	3.330 (4)	144

Symmetry code: (i) 
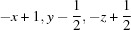
.

**Fig. 1.**
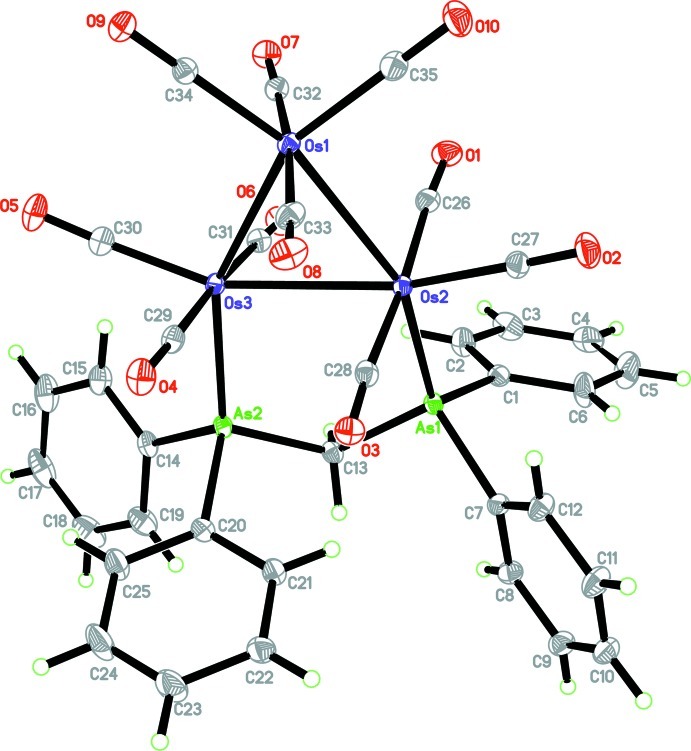
The molecular structure of the title compound showing 30% probability displacement ellipsoids for non-H atoms.

**Fig. 2.**
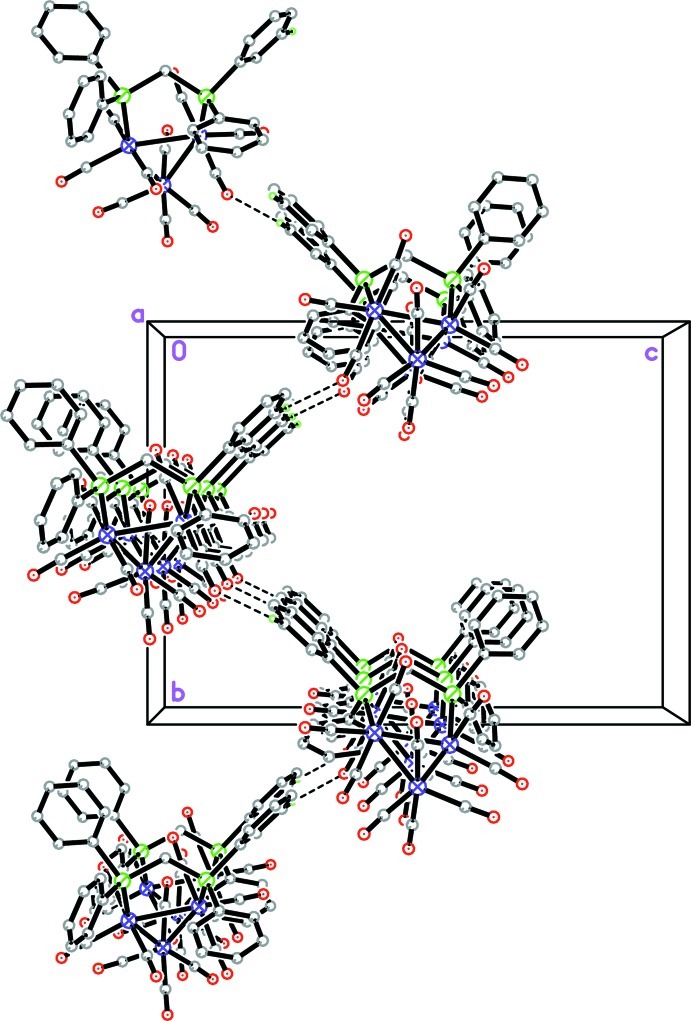
The crystal structure of the title compound, viewed along the a axis. H atoms not involved in hydrogen bonds (dashed lines) have been omitted for clarity.

## supplementary crystallographic information

### Comment

A large number of substituted derivatives of the type *M*_3_(CO)_12-_*_n_L_n_* (*M* = Ru, Os and *L* = group 15 ligand) have been reported (Bruce *et al.*,1985, 1988*a*,*b*). As part of our study on the substitution of transition metal-carbonyl clusters with mixed-ligand complexes, we have published several structures of *triangulo*-triruthenium-carbonyl clusters containing mixed P/As and P/Sb ligands(Shawkataly *et al.*, 1998, 2004, 2010). Herein we report the synthesis and structure of the title compound.

The title *triangulo*-triosmium compound, Fig. 1, contains a triangle of singly bonded Os atoms. The bis(diphenylarsino)methane ligand bridges the Os2–Os3 bond. Both arsine ligands are equatorial with respect to the Os_3_ triangle. Additionally, the Os1 atom carries two equatorial and two axial terminal carbonyl ligands whereas the Os2 and Os3 atoms each carries one equatorial and two axial terminal carbonyl ligands (Fig. 1). The dihedral angles between the two benzene rings (C1–C6/C7–C12 and C14–C19/C20–C25) are 67.42 (16) and 61.99 (16)° for the two diphenylarsino groups respectively.

In the crystal structure, Fig. 2, molecules are linked *via* intermolecular C16–H16A···O4 hydrogen bonds (Table 1) into one-dimensional zig-zag chains along [010].

### Experimental

All manipulations were performed under a dry oxygen-free nitrogen atmosphere using standard Schlenk techniques. All solvents were dried over sodium and distilled from sodium benzophenone ketyl under dry oxygen free nitrogen. The title compound was obtained by refluxing equimolar quantities of Os_3_(CO)_12_ and (C_6_H_5_)_2_AsCH_2_As(C_6_H_5_)_2_ in methanol under nitrogen atmosphere. Yellow blocks were grown by slow solvent/solvent diffusion of CH_3_OH into CH_2_Cl_2_.

### Refinement

All H atoms were positioned geometrically and refined using a riding model with C–H = 0.93 or 0.97 Å and *U*_iso_(H) = 1.2 *U*_eq_(C).

### Crystal data

**Table:**

[Os_3_(C_25_H_22_As_2_)(CO)_10_]	*F*(000) = 2424
*M**_r_* = 1322.97	*D*_x_ = 2.451 Mg m^−^^3^
Orthorhombic, *P*2_1_2_1_2_1_	Mo *K*α radiation, λ = 0.71073 Å
Hall symbol: P 2ac 2ab	Cell parameters from 9235 reflections
*a* = 11.2965 (4) Å	θ = 3.5–35.0°
*b* = 15.3594 (5) Å	µ = 12.49 mm^−^^1^
*c* = 20.6641 (7) Å	*T* = 100 K
*V* = 3585.4 (2) Å^3^	Block, yellow
*Z* = 4	0.28 × 0.19 × 0.08 mm

### Data collection

**Table:**

Bruker SMART APEXII DUO CCD diffractometer	15930 independent reflections
Radiation source: fine-focus sealed tube	15315 reflections with *I* > 2σ(*I*)
Graphite monochromator	*R*_int_ = 0.038
φ and ω scans	θ_max_ = 35.2°, θ_min_ = 1.7°
Absorption correction: multi-scan (*SADABS*; Bruker, 2009)	*h* = −18→17
*T*_min_ = 0.126, *T*_max_ = 0.424	*k* = −24→24
113235 measured reflections	*l* = −33→31

### Refinement

**Table:**

Refinement on *F*^2^	Secondary atom site location: difference Fourier map
Least-squares matrix: full	Hydrogen site location: inferred from neighbouring sites
*R*[*F*^2^ > 2σ(*F*^2^)] = 0.018	H-atom parameters constrained
*wR*(*F*^2^) = 0.040	*w* = 1/[σ^2^(*F*_o_^2^) + (0.0183*P*)^2^ + 0.1317*P*] where *P* = (*F*_o_^2^ + 2*F*_c_^2^)/3
*S* = 1.03	(Δ/σ)_max_ = 0.004
15930 reflections	Δρ_max_ = 2.15 e Å^−^^3^
451 parameters	Δρ_min_ = −0.66 e Å^−^^3^
0 restraints	Absolute structure: Flack (1983), 7211 Friedel pairs
Primary atom site location: structure-invariant direct methods	Flack parameter: 0.003 (4)

### Special details

**Table:**

Experimental. The crystal was placed in the cold stream of an Oxford Cryosystems Cobra open-flow nitrogen cryostat (Cosier & Glazer, 1986) operating at 100 K.
Geometry. All esds (except the esd in the dihedral angle between two l.s. planes) are estimated using the full covariance matrix. The cell esds are taken into account individually in the estimation of esds in distances, angles and torsion angles; correlations between esds in cell parameters are only used when they are defined by crystal symmetry. An approximate (isotropic) treatment of cell esds is used for estimating esds involving l.s. planes.
Refinement. Refinement of F^2^ against ALL reflections. The weighted R-factor wR and goodness of fit S are based on F^2^, conventional R-factors R are based on F, with F set to zero for negative F^2^. The threshold expression of F^2^ > 2sigma(F^2^) is used only for calculating R-factors(gt) etc. and is not relevant to the choice of reflections for refinement. R-factors based on F^2^ are statistically about twice as large as those based on F, and R- factors based on ALL data will be even larger.

### Fractional atomic coordinates and isotropic or equivalent isotropic displacement parameters (Å^2^)

**Table:**

	*x*	*y*	*z*	*U*_iso_*/*U*_eq_	
Os1	0.354069 (9)	0.615329 (6)	0.007987 (5)	0.01876 (2)	
Os2	0.548452 (9)	0.526643 (6)	−0.050964 (4)	0.01714 (2)	
Os3	0.489703 (8)	0.495413 (6)	0.082197 (4)	0.01706 (2)	
As1	0.69820 (2)	0.412545 (15)	−0.057934 (12)	0.01689 (4)	
As2	0.67020 (2)	0.413355 (15)	0.100713 (12)	0.01737 (4)	
O1	0.3597 (2)	0.40377 (15)	−0.10762 (11)	0.0328 (5)	
O2	0.5677 (2)	0.62136 (16)	−0.17955 (10)	0.0362 (5)	
O3	0.73319 (18)	0.65213 (12)	0.00711 (10)	0.0249 (4)	
O4	0.6145 (2)	0.65723 (13)	0.13870 (11)	0.0314 (5)	
O5	0.3398 (2)	0.47636 (15)	0.20337 (11)	0.0364 (5)	
O6	0.3869 (2)	0.32433 (13)	0.02838 (11)	0.0309 (4)	
O7	0.1858 (2)	0.45691 (13)	0.01110 (11)	0.0290 (4)	
O8	0.5231 (2)	0.77265 (13)	0.02219 (12)	0.0330 (5)	
O9	0.1940 (2)	0.71457 (14)	0.10128 (10)	0.0274 (4)	
O10	0.2597 (3)	0.67957 (16)	−0.12203 (11)	0.0375 (5)	
C1	0.6686 (2)	0.31404 (16)	−0.11490 (13)	0.0209 (4)	
C2	0.6021 (3)	0.24306 (19)	−0.09540 (15)	0.0306 (6)	
H2A	0.5695	0.2419	−0.0541	0.037*	
C3	0.5837 (3)	0.1734 (2)	−0.13716 (18)	0.0357 (7)	
H3A	0.5414	0.1249	−0.1233	0.043*	
C4	0.6283 (3)	0.1764 (2)	−0.19913 (18)	0.0387 (8)	
H4A	0.6156	0.1302	−0.2273	0.046*	
C5	0.6920 (4)	0.2480 (3)	−0.21935 (19)	0.0507 (11)	
H5A	0.7204	0.2504	−0.2615	0.061*	
C6	0.7138 (4)	0.3161 (3)	−0.17744 (15)	0.0393 (8)	
H6A	0.7587	0.3635	−0.1911	0.047*	
C7	0.8535 (2)	0.44692 (16)	−0.08874 (12)	0.0195 (4)	
C8	0.9464 (2)	0.38678 (17)	−0.08878 (13)	0.0239 (4)	
H8A	0.9348	0.3316	−0.0714	0.029*	
C9	1.0558 (3)	0.40890 (19)	−0.11464 (14)	0.0277 (5)	
H9A	1.1176	0.3688	−0.1143	0.033*	
C10	1.0728 (3)	0.4917 (2)	−0.14121 (14)	0.0293 (5)	
H10A	1.1456	0.5067	−0.1590	0.035*	
C11	0.9808 (3)	0.5514 (2)	−0.14105 (16)	0.0319 (6)	
H11A	0.9928	0.6066	−0.1584	0.038*	
C12	0.8710 (3)	0.53001 (18)	−0.11529 (14)	0.0256 (5)	
H12A	0.8096	0.5704	−0.1156	0.031*	
C13	0.7304 (2)	0.35167 (16)	0.02453 (12)	0.0200 (4)	
H13A	0.6947	0.2943	0.0229	0.024*	
H13B	0.8152	0.3440	0.0291	0.024*	
C14	0.6633 (3)	0.32178 (16)	0.16483 (12)	0.0226 (5)	
C15	0.5550 (3)	0.30088 (18)	0.19310 (14)	0.0287 (5)	
H15A	0.4860	0.3283	0.1793	0.034*	
C16	0.5504 (4)	0.2387 (2)	0.24227 (16)	0.0406 (8)	
H16A	0.4785	0.2253	0.2619	0.049*	
C17	0.6525 (5)	0.1971 (2)	0.26166 (16)	0.0448 (10)	
H17A	0.6493	0.1558	0.2945	0.054*	
C18	0.7609 (4)	0.2164 (2)	0.23230 (19)	0.0455 (9)	
H18A	0.8292	0.1875	0.2452	0.055*	
C19	0.7663 (3)	0.2790 (2)	0.18377 (17)	0.0348 (7)	
H19A	0.8382	0.2921	0.1641	0.042*	
C20	0.8073 (2)	0.47859 (17)	0.12749 (12)	0.0218 (4)	
C21	0.8823 (2)	0.51501 (17)	0.08151 (13)	0.0235 (5)	
H21A	0.8685	0.5044	0.0379	0.028*	
C22	0.9774 (3)	0.5669 (2)	0.09953 (15)	0.0295 (6)	
H22A	1.0258	0.5924	0.0684	0.035*	
C23	0.9990 (4)	0.5801 (3)	0.16520 (17)	0.0416 (8)	
H23A	1.0631	0.6139	0.1781	0.050*	
C24	0.9261 (4)	0.5433 (3)	0.21142 (16)	0.0442 (9)	
H24A	0.9419	0.5521	0.2551	0.053*	
C25	0.8293 (3)	0.4932 (2)	0.19326 (14)	0.0332 (6)	
H25A	0.7795	0.4696	0.2245	0.040*	
C26	0.4277 (2)	0.44858 (18)	−0.08441 (14)	0.0246 (5)	
C27	0.5611 (3)	0.58390 (18)	−0.13190 (13)	0.0249 (5)	
C28	0.6617 (2)	0.60593 (16)	−0.01254 (12)	0.0213 (4)	
C29	0.5671 (2)	0.59914 (16)	0.11534 (12)	0.0222 (5)	
C30	0.3988 (3)	0.48449 (17)	0.15851 (14)	0.0254 (5)	
C31	0.4223 (2)	0.39043 (17)	0.04611 (13)	0.0233 (4)	
C32	0.2519 (2)	0.51294 (17)	0.00885 (13)	0.0235 (4)	
C33	0.4661 (3)	0.71218 (17)	0.01634 (14)	0.0257 (5)	
C34	0.2522 (2)	0.67485 (16)	0.06705 (13)	0.0217 (4)	
C35	0.2952 (3)	0.65769 (18)	−0.07368 (15)	0.0274 (5)	

### Atomic displacement parameters (Å^2^)

**Table:**

	*U*^11^	*U*^22^	*U*^33^	*U*^12^	*U*^13^	*U*^23^
Os1	0.01912 (4)	0.01713 (4)	0.02001 (4)	0.00236 (3)	0.00210 (3)	0.00109 (3)
Os2	0.01921 (4)	0.01654 (3)	0.01566 (3)	0.00183 (3)	0.00101 (3)	0.00057 (3)
Os3	0.01947 (4)	0.01557 (3)	0.01613 (3)	−0.00027 (3)	0.00148 (3)	0.00011 (3)
As1	0.01900 (11)	0.01576 (9)	0.01591 (10)	0.00097 (8)	0.00047 (8)	−0.00040 (8)
As2	0.02087 (11)	0.01588 (9)	0.01538 (9)	−0.00059 (8)	−0.00076 (8)	0.00113 (8)
O1	0.0256 (10)	0.0353 (11)	0.0376 (11)	−0.0034 (8)	−0.0039 (9)	−0.0105 (9)
O2	0.0463 (14)	0.0400 (12)	0.0223 (9)	0.0023 (10)	0.0011 (9)	0.0086 (9)
O3	0.0247 (9)	0.0211 (8)	0.0289 (9)	−0.0023 (7)	−0.0005 (8)	0.0011 (7)
O4	0.0421 (13)	0.0218 (9)	0.0302 (10)	−0.0078 (8)	0.0055 (9)	−0.0044 (8)
O5	0.0473 (14)	0.0326 (10)	0.0291 (10)	−0.0087 (10)	0.0161 (10)	−0.0033 (8)
O6	0.0379 (12)	0.0208 (9)	0.0340 (11)	−0.0038 (8)	−0.0040 (9)	−0.0028 (8)
O7	0.0289 (10)	0.0261 (9)	0.0320 (10)	−0.0033 (7)	−0.0008 (8)	−0.0011 (8)
O8	0.0293 (11)	0.0232 (9)	0.0466 (13)	−0.0018 (8)	0.0053 (9)	0.0033 (9)
O9	0.0274 (10)	0.0280 (9)	0.0267 (9)	0.0010 (8)	0.0051 (8)	−0.0040 (8)
O10	0.0499 (15)	0.0369 (12)	0.0259 (10)	0.0080 (11)	−0.0017 (10)	0.0050 (9)
C1	0.0206 (11)	0.0212 (10)	0.0209 (10)	0.0028 (8)	−0.0013 (8)	−0.0024 (8)
C2	0.0379 (16)	0.0267 (12)	0.0273 (13)	−0.0095 (11)	−0.0011 (11)	−0.0033 (10)
C3	0.0417 (18)	0.0224 (12)	0.0429 (17)	−0.0044 (12)	−0.0107 (14)	−0.0047 (12)
C4	0.0351 (17)	0.0377 (16)	0.0432 (18)	0.0033 (13)	−0.0091 (14)	−0.0208 (14)
C5	0.055 (2)	0.067 (3)	0.0296 (16)	−0.020 (2)	0.0093 (16)	−0.0261 (17)
C6	0.050 (2)	0.0475 (18)	0.0200 (12)	−0.0189 (16)	0.0031 (13)	−0.0073 (12)
C7	0.0181 (10)	0.0220 (10)	0.0183 (10)	0.0002 (8)	0.0007 (8)	−0.0029 (8)
C8	0.0219 (11)	0.0227 (10)	0.0271 (11)	0.0021 (9)	0.0003 (9)	−0.0009 (9)
C9	0.0227 (12)	0.0320 (13)	0.0284 (12)	0.0013 (10)	0.0026 (10)	−0.0049 (10)
C10	0.0245 (12)	0.0354 (14)	0.0281 (12)	−0.0037 (11)	0.0056 (10)	0.0003 (11)
C11	0.0318 (15)	0.0296 (13)	0.0345 (14)	−0.0037 (11)	0.0096 (12)	0.0076 (11)
C12	0.0259 (13)	0.0224 (11)	0.0286 (12)	0.0003 (9)	0.0062 (10)	0.0028 (9)
C13	0.0242 (11)	0.0173 (9)	0.0183 (10)	0.0036 (8)	−0.0029 (9)	−0.0003 (8)
C14	0.0323 (14)	0.0186 (10)	0.0168 (10)	−0.0017 (9)	−0.0016 (9)	0.0012 (8)
C15	0.0397 (16)	0.0219 (11)	0.0246 (12)	−0.0032 (11)	0.0047 (11)	0.0047 (9)
C16	0.068 (2)	0.0280 (13)	0.0260 (13)	−0.0133 (15)	0.0066 (15)	0.0055 (11)
C17	0.082 (3)	0.0273 (14)	0.0254 (13)	−0.0134 (17)	−0.0116 (17)	0.0092 (11)
C18	0.059 (2)	0.0367 (17)	0.0408 (19)	−0.0009 (16)	−0.0207 (18)	0.0140 (14)
C19	0.0407 (18)	0.0298 (14)	0.0340 (15)	0.0022 (12)	−0.0099 (13)	0.0100 (12)
C20	0.0229 (11)	0.0216 (10)	0.0209 (10)	−0.0010 (8)	−0.0027 (8)	−0.0003 (8)
C21	0.0268 (12)	0.0236 (11)	0.0201 (10)	−0.0026 (9)	−0.0017 (9)	−0.0002 (9)
C22	0.0259 (13)	0.0337 (13)	0.0290 (13)	−0.0074 (10)	−0.0022 (10)	0.0022 (11)
C23	0.0418 (18)	0.0499 (19)	0.0332 (15)	−0.0222 (16)	−0.0085 (14)	−0.0006 (14)
C24	0.054 (2)	0.054 (2)	0.0238 (13)	−0.0253 (18)	−0.0113 (14)	−0.0015 (13)
C25	0.0414 (17)	0.0374 (15)	0.0207 (11)	−0.0138 (13)	−0.0028 (11)	−0.0006 (11)
C26	0.0235 (12)	0.0262 (11)	0.0241 (11)	0.0013 (9)	0.0011 (10)	−0.0021 (10)
C27	0.0258 (12)	0.0270 (12)	0.0217 (11)	0.0028 (10)	0.0017 (9)	−0.0014 (9)
C28	0.0253 (11)	0.0195 (10)	0.0191 (10)	0.0046 (8)	0.0012 (9)	0.0023 (8)
C29	0.0263 (12)	0.0208 (10)	0.0196 (10)	−0.0013 (9)	0.0052 (9)	0.0010 (8)
C30	0.0300 (13)	0.0191 (11)	0.0270 (12)	−0.0021 (9)	0.0039 (10)	−0.0013 (9)
C31	0.0233 (11)	0.0215 (10)	0.0252 (11)	0.0004 (8)	0.0003 (9)	0.0006 (9)
C32	0.0240 (11)	0.0256 (11)	0.0209 (10)	0.0033 (9)	0.0006 (9)	−0.0016 (9)
C33	0.0248 (12)	0.0223 (10)	0.0300 (13)	0.0029 (9)	0.0054 (10)	0.0026 (9)
C34	0.0229 (11)	0.0211 (10)	0.0211 (11)	−0.0015 (9)	0.0004 (9)	0.0021 (8)
C35	0.0316 (14)	0.0225 (11)	0.0281 (13)	0.0068 (10)	0.0053 (11)	0.0039 (10)

### Geometric parameters (Å, º)

**Table:**

Os1—C34	1.910 (3)	C5—C6	1.380 (5)
Os1—C35	1.927 (3)	C5—H5A	0.9300
Os1—C32	1.951 (3)	C6—H6A	0.9300
Os1—C33	1.961 (3)	C7—C8	1.397 (4)
Os1—Os3	2.8446 (2)	C7—C12	1.403 (4)
Os1—Os2	2.8568 (2)	C8—C9	1.389 (4)
Os2—C27	1.895 (3)	C8—H8A	0.9300
Os2—C28	1.937 (3)	C9—C10	1.398 (4)
Os2—C26	1.943 (3)	C9—H9A	0.9300
Os2—As1	2.4400 (3)	C10—C11	1.386 (4)
Os2—Os3	2.8709 (2)	C10—H10A	0.9300
Os3—C30	1.889 (3)	C11—C12	1.390 (4)
Os3—C31	1.933 (3)	C11—H11A	0.9300
Os3—C29	1.942 (3)	C12—H12A	0.9300
Os3—As2	2.4274 (3)	C13—H13A	0.9700
As1—C7	1.940 (3)	C13—H13B	0.9700
As1—C1	1.946 (3)	C14—C19	1.393 (4)
As1—C13	1.977 (2)	C14—C15	1.393 (4)
As2—C20	1.926 (3)	C15—C16	1.396 (4)
As2—C14	1.934 (2)	C15—H15A	0.9300
As2—C13	1.959 (2)	C16—C17	1.378 (6)
O1—C26	1.138 (3)	C16—H16A	0.9300
O2—C27	1.143 (3)	C17—C18	1.398 (7)
O3—C28	1.149 (3)	C17—H17A	0.9300
O4—C29	1.147 (3)	C18—C19	1.390 (5)
O5—C30	1.149 (3)	C18—H18A	0.9300
O6—C31	1.151 (3)	C19—H19A	0.9300
O7—C32	1.140 (3)	C20—C21	1.390 (4)
O8—C33	1.137 (3)	C20—C25	1.400 (4)
O9—C34	1.142 (3)	C21—C22	1.389 (4)
O10—C35	1.128 (4)	C21—H21A	0.9300
C1—C2	1.384 (4)	C22—C23	1.393 (4)
C1—C6	1.390 (4)	C22—H22A	0.9300
C2—C3	1.390 (4)	C23—C24	1.383 (5)
C2—H2A	0.9300	C23—H23A	0.9300
C3—C4	1.377 (5)	C24—C25	1.389 (5)
C3—H3A	0.9300	C24—H24A	0.9300
C4—C5	1.378 (6)	C25—H25A	0.9300
C4—H4A	0.9300		
			
C34—Os1—C35	100.97 (12)	C5—C6—H6A	120.0
C34—Os1—C32	91.35 (11)	C1—C6—H6A	120.0
C35—Os1—C32	94.37 (12)	C8—C7—C12	119.7 (2)
C34—Os1—C33	88.26 (11)	C8—C7—As1	119.94 (19)
C35—Os1—C33	92.50 (12)	C12—C7—As1	120.2 (2)
C32—Os1—C33	173.07 (12)	C9—C8—C7	120.4 (2)
C34—Os1—Os3	106.85 (8)	C9—C8—H8A	119.8
C35—Os1—Os3	151.23 (8)	C7—C8—H8A	119.8
C32—Os1—Os3	77.99 (8)	C8—C9—C10	119.7 (3)
C33—Os1—Os3	95.51 (8)	C8—C9—H9A	120.2
C34—Os1—Os2	164.49 (8)	C10—C9—H9A	120.2
C35—Os1—Os2	93.02 (8)	C11—C10—C9	119.9 (3)
C32—Os1—Os2	94.26 (8)	C11—C10—H10A	120.1
C33—Os1—Os2	84.43 (8)	C9—C10—H10A	120.1
Os3—Os1—Os2	60.470 (4)	C10—C11—C12	120.9 (3)
C27—Os2—C28	91.16 (11)	C10—C11—H11A	119.5
C27—Os2—C26	91.45 (12)	C12—C11—H11A	119.5
C28—Os2—C26	176.04 (11)	C11—C12—C7	119.4 (3)
C27—Os2—As1	103.24 (8)	C11—C12—H12A	120.3
C28—Os2—As1	91.01 (7)	C7—C12—H12A	120.3
C26—Os2—As1	91.29 (8)	As2—C13—As1	113.57 (12)
C27—Os2—Os1	102.30 (8)	As2—C13—H13A	108.9
C28—Os2—Os1	91.91 (8)	As1—C13—H13A	108.9
C26—Os2—Os1	84.62 (8)	As2—C13—H13B	108.9
As1—Os2—Os1	154.228 (7)	As1—C13—H13B	108.9
C27—Os2—Os3	160.20 (8)	H13A—C13—H13B	107.7
C28—Os2—Os3	82.24 (7)	C19—C14—C15	120.5 (3)
C26—Os2—Os3	94.33 (8)	C19—C14—As2	120.1 (2)
As1—Os2—Os3	95.557 (7)	C15—C14—As2	119.4 (2)
Os1—Os2—Os3	59.555 (3)	C14—C15—C16	119.7 (3)
C30—Os3—C31	91.94 (11)	C14—C15—H15A	120.1
C30—Os3—C29	91.33 (11)	C16—C15—H15A	120.1
C31—Os3—C29	176.16 (11)	C17—C16—C15	119.9 (4)
C30—Os3—As2	106.18 (9)	C17—C16—H16A	120.1
C31—Os3—As2	87.62 (8)	C15—C16—H16A	120.1
C29—Os3—As2	89.54 (8)	C16—C17—C18	120.6 (3)
C30—Os3—Os1	102.41 (9)	C16—C17—H17A	119.7
C31—Os3—Os1	96.89 (8)	C18—C17—H17A	119.7
C29—Os3—Os1	84.35 (8)	C19—C18—C17	119.9 (4)
As2—Os3—Os1	150.881 (7)	C19—C18—H18A	120.0
C30—Os3—Os2	160.15 (9)	C17—C18—H18A	120.0
C31—Os3—Os2	81.99 (8)	C18—C19—C14	119.4 (4)
C29—Os3—Os2	95.56 (7)	C18—C19—H19A	120.3
As2—Os3—Os2	92.502 (7)	C14—C19—H19A	120.3
Os1—Os3—Os2	59.976 (4)	C21—C20—C25	119.4 (3)
C7—As1—C1	99.71 (10)	C21—C20—As2	120.20 (19)
C7—As1—C13	104.18 (11)	C25—C20—As2	120.3 (2)
C1—As1—C13	100.68 (10)	C22—C21—C20	121.3 (3)
C7—As1—Os2	116.81 (7)	C22—C21—H21A	119.4
C1—As1—Os2	118.35 (8)	C20—C21—H21A	119.4
C13—As1—Os2	114.60 (7)	C21—C22—C23	118.7 (3)
C20—As2—C14	102.36 (11)	C21—C22—H22A	120.7
C20—As2—C13	101.73 (11)	C23—C22—H22A	120.7
C14—As2—C13	102.29 (11)	C24—C23—C22	120.6 (3)
C20—As2—Os3	116.79 (8)	C24—C23—H23A	119.7
C14—As2—Os3	116.85 (9)	C22—C23—H23A	119.7
C13—As2—Os3	114.59 (7)	C23—C24—C25	120.6 (3)
C2—C1—C6	119.2 (3)	C23—C24—H24A	119.7
C2—C1—As1	122.0 (2)	C25—C24—H24A	119.7
C6—C1—As1	118.8 (2)	C24—C25—C20	119.4 (3)
C1—C2—C3	120.4 (3)	C24—C25—H25A	120.3
C1—C2—H2A	119.8	C20—C25—H25A	120.3
C3—C2—H2A	119.8	O1—C26—Os2	175.9 (3)
C4—C3—C2	119.8 (3)	O2—C27—Os2	177.4 (3)
C4—C3—H3A	120.1	O3—C28—Os2	175.9 (2)
C2—C3—H3A	120.1	O4—C29—Os3	175.3 (2)
C3—C4—C5	120.0 (3)	O5—C30—Os3	177.1 (3)
C3—C4—H4A	120.0	O6—C31—Os3	174.6 (2)
C5—C4—H4A	120.0	O7—C32—Os1	175.0 (2)
C4—C5—C6	120.5 (3)	O8—C33—Os1	174.2 (2)
C4—C5—H5A	119.8	O9—C34—Os1	176.3 (2)
C6—C5—H5A	119.8	O10—C35—Os1	177.6 (3)
C5—C6—C1	120.0 (3)		
			
C34—Os1—Os2—C27	−134.2 (3)	C27—Os2—As1—C13	169.69 (12)
C35—Os1—Os2—C27	20.33 (13)	C28—Os2—As1—C13	78.26 (11)
C32—Os1—Os2—C27	114.94 (12)	C26—Os2—As1—C13	−98.51 (12)
C33—Os1—Os2—C27	−71.89 (12)	Os1—Os2—As1—C13	−18.24 (9)
Os3—Os1—Os2—C27	−171.44 (9)	Os3—Os2—As1—C13	−4.03 (8)
C34—Os1—Os2—C28	−42.5 (3)	C30—Os3—As2—C20	−97.89 (12)
C35—Os1—Os2—C28	111.94 (12)	C31—Os3—As2—C20	170.77 (12)
C32—Os1—Os2—C28	−153.45 (11)	C29—Os3—As2—C20	−6.64 (11)
C33—Os1—Os2—C28	19.72 (11)	Os1—Os3—As2—C20	70.85 (9)
Os3—Os1—Os2—C28	−79.84 (7)	Os2—Os3—As2—C20	88.90 (9)
C34—Os1—Os2—C26	135.5 (3)	C30—Os3—As2—C14	23.73 (12)
C35—Os1—Os2—C26	−69.98 (12)	C31—Os3—As2—C14	−67.60 (12)
C32—Os1—Os2—C26	24.63 (12)	C29—Os3—As2—C14	114.98 (11)
C33—Os1—Os2—C26	−162.20 (12)	Os1—Os3—As2—C14	−167.53 (8)
Os3—Os1—Os2—C26	98.24 (8)	Os2—Os3—As2—C14	−149.47 (9)
C34—Os1—Os2—As1	53.7 (3)	C30—Os3—As2—C13	143.33 (12)
C35—Os1—Os2—As1	−151.77 (9)	C31—Os3—As2—C13	52.00 (12)
C32—Os1—Os2—As1	−57.16 (8)	C29—Os3—As2—C13	−125.42 (11)
C33—Os1—Os2—As1	116.01 (9)	Os1—Os3—As2—C13	−47.93 (9)
Os3—Os1—Os2—As1	16.456 (16)	Os2—Os3—As2—C13	−29.87 (8)
C34—Os1—Os2—Os3	37.3 (3)	C7—As1—C1—C2	148.3 (2)
C35—Os1—Os2—Os3	−168.22 (9)	C13—As1—C1—C2	41.7 (3)
C32—Os1—Os2—Os3	−73.61 (8)	Os2—As1—C1—C2	−83.9 (2)
C33—Os1—Os2—Os3	99.56 (9)	C7—As1—C1—C6	−33.0 (3)
C34—Os1—Os3—C30	19.63 (12)	C13—As1—C1—C6	−139.5 (3)
C35—Os1—Os3—C30	−145.1 (2)	Os2—As1—C1—C6	94.8 (3)
C32—Os1—Os3—C30	−68.12 (12)	C6—C1—C2—C3	1.9 (5)
C33—Os1—Os3—C30	109.47 (12)	As1—C1—C2—C3	−179.4 (3)
Os2—Os1—Os3—C30	−170.12 (8)	C1—C2—C3—C4	−2.3 (5)
C34—Os1—Os3—C31	113.16 (12)	C2—C3—C4—C5	0.6 (6)
C35—Os1—Os3—C31	−51.5 (2)	C3—C4—C5—C6	1.5 (7)
C32—Os1—Os3—C31	25.41 (11)	C4—C5—C6—C1	−1.9 (7)
C33—Os1—Os3—C31	−157.00 (11)	C2—C1—C6—C5	0.2 (6)
Os2—Os1—Os3—C31	−76.59 (8)	As1—C1—C6—C5	−178.6 (3)
C34—Os1—Os3—C29	−70.49 (11)	C1—As1—C7—C8	−56.7 (2)
C35—Os1—Os3—C29	124.8 (2)	C13—As1—C7—C8	47.0 (2)
C32—Os1—Os3—C29	−158.24 (11)	Os2—As1—C7—C8	174.55 (18)
C33—Os1—Os3—C29	19.35 (11)	C1—As1—C7—C12	118.8 (2)
Os2—Os1—Os3—C29	99.76 (8)	C13—As1—C7—C12	−137.5 (2)
C34—Os1—Os3—As2	−149.30 (8)	Os2—As1—C7—C12	−10.0 (2)
C35—Os1—Os3—As2	46.00 (19)	C12—C7—C8—C9	0.3 (4)
C32—Os1—Os3—As2	122.95 (8)	As1—C7—C8—C9	175.8 (2)
C33—Os1—Os3—As2	−59.46 (8)	C7—C8—C9—C10	−0.5 (4)
Os2—Os1—Os3—As2	20.951 (15)	C8—C9—C10—C11	0.6 (4)
C34—Os1—Os3—Os2	−170.25 (8)	C9—C10—C11—C12	−0.6 (5)
C35—Os1—Os3—Os2	25.05 (19)	C10—C11—C12—C7	0.4 (5)
C32—Os1—Os3—Os2	102.00 (8)	C8—C7—C12—C11	−0.3 (4)
C33—Os1—Os3—Os2	−80.41 (8)	As1—C7—C12—C11	−175.7 (2)
C27—Os2—Os3—C30	55.0 (4)	C20—As2—C13—As1	−94.37 (14)
C28—Os2—Os3—C30	126.4 (2)	C14—As2—C13—As1	160.03 (13)
C26—Os2—Os3—C30	−51.6 (2)	Os3—As2—C13—As1	32.59 (15)
As1—Os2—Os3—C30	−143.3 (2)	C7—As1—C13—As2	113.07 (14)
Os1—Os2—Os3—C30	29.6 (2)	C1—As1—C13—As2	−143.94 (13)
C27—Os2—Os3—C31	128.2 (3)	Os2—As1—C13—As2	−15.79 (16)
C28—Os2—Os3—C31	−160.37 (11)	C20—As2—C14—C19	−43.3 (3)
C26—Os2—Os3—C31	21.62 (11)	C13—As2—C14—C19	61.8 (3)
As1—Os2—Os3—C31	−70.11 (8)	Os3—As2—C14—C19	−172.2 (2)
Os1—Os2—Os3—C31	102.78 (8)	C20—As2—C14—C15	134.9 (2)
C27—Os2—Os3—C29	−54.8 (3)	C13—As2—C14—C15	−120.0 (2)
C28—Os2—Os3—C29	16.66 (11)	Os3—As2—C14—C15	6.0 (2)
C26—Os2—Os3—C29	−161.36 (11)	C19—C14—C15—C16	2.2 (4)
As1—Os2—Os3—C29	106.92 (8)	As2—C14—C15—C16	−176.0 (2)
Os1—Os2—Os3—C29	−80.19 (8)	C14—C15—C16—C17	−1.3 (5)
C27—Os2—Os3—As2	−144.6 (3)	C15—C16—C17—C18	−0.2 (5)
C28—Os2—Os3—As2	−73.13 (7)	C16—C17—C18—C19	0.9 (6)
C26—Os2—Os3—As2	108.86 (8)	C17—C18—C19—C14	−0.1 (5)
As1—Os2—Os3—As2	17.139 (9)	C15—C14—C19—C18	−1.5 (5)
Os1—Os2—Os3—As2	−169.970 (7)	As2—C14—C19—C18	176.7 (3)
C27—Os2—Os3—Os1	25.4 (3)	C14—As2—C20—C21	143.4 (2)
C28—Os2—Os3—Os1	96.84 (7)	C13—As2—C20—C21	37.8 (2)
C26—Os2—Os3—Os1	−81.17 (8)	Os3—As2—C20—C21	−87.7 (2)
As1—Os2—Os3—Os1	−172.891 (7)	C14—As2—C20—C25	−39.6 (3)
C27—Os2—As1—C7	47.46 (12)	C13—As2—C20—C25	−145.1 (2)
C28—Os2—As1—C7	−43.97 (11)	Os3—As2—C20—C25	89.3 (2)
C26—Os2—As1—C7	139.26 (11)	C25—C20—C21—C22	−1.2 (4)
Os1—Os2—As1—C7	−140.47 (8)	As2—C20—C21—C22	175.9 (2)
Os3—Os2—As1—C7	−126.26 (8)	C20—C21—C22—C23	1.9 (5)
C27—Os2—As1—C1	−71.71 (13)	C21—C22—C23—C24	−1.0 (6)
C28—Os2—As1—C1	−163.14 (12)	C22—C23—C24—C25	−0.6 (7)
C26—Os2—As1—C1	20.08 (12)	C23—C24—C25—C20	1.3 (6)
Os1—Os2—As1—C1	100.36 (9)	C21—C20—C25—C24	−0.5 (5)
Os3—Os2—As1—C1	114.56 (9)	As2—C20—C25—C24	−177.5 (3)

### Hydrogen-bond geometry (Å, º)

**Table:**

*D*—H···*A*	*D*—H	H···*A*	*D*···*A*	*D*—H···*A*
C16—H16*A*···O4^i^	0.93	2.53	3.330 (4)	144

Symmetry code: (i) −*x*+1, *y*−1/2, −*z*+1/2.
